# Organoid models of the pituitary gland in health and disease

**DOI:** 10.3389/fendo.2023.1233714

**Published:** 2023-08-08

**Authors:** Emma Laporte, Hugo Vankelecom

**Affiliations:** Department of Development and Regeneration, Cluster of Stem Cell and Developmental Biology, Laboratory of Tissue Plasticity in Health and Disease, Katholieke Universiteit (KU) Leuven, Leuven, Belgium

**Keywords:** pituitary, organoids, pituitary stem cells, pluripotent stem cells, PitNETs

## Abstract

The pituitary gland represents the hub of our endocrine system. Its cells produce specific hormones that direct multiple vital physiological processes such as body growth, fertility, and stress. The gland also contains a population of stem cells which are still enigmatic in phenotype and function. Appropriate research models are needed to advance our knowledge on pituitary (stem cell) biology. Over the last decade, 3D organoid models have been established, either derived from the pituitary stem cells or from pluripotent stem cells, covering both healthy and diseased conditions. Here, we summarize the state-of-the-art of pituitary-allied organoid models and discuss applications of these powerful *in vitro* research and translational tools to study pituitary development, biology, and disease.

## 1 Introduction

### 1.1 The pituitary gland

#### 1.1.1 Anatomy and functionality

The pituitary gland is a key player in the endocrine system. Together with the hypothalamus, it governs the physiological processes of, among others, body growth, metabolism, reproduction, lactation, and stress. The mouse pituitary consists of three segments, encompassing the anterior, intermediate, and posterior lobe (AL, IL, and PL, respectively). In some mammals including humans, the IL is not present anymore as separate entity but its endocrine cells, the melanocyte-stimulating hormone (MSH)-producing melanotropes, have become part of the AL. Primarily, the AL is composed of specific hormone-producing cell types, i.e., somatotropes that produce growth hormone (GH), lactotropes that make prolactin (PRL), corticotropes that secrete adrenocorticotropic hormone (ACTH), thyrotropes that produce thyroid-stimulating hormone (TSH), and gonadotropes that generate luteinizing hormone (LH) and/or follicle-stimulating hormone (FSH). These trophic hormones stimulate the release of hormones from target organs including the adrenal cortex, gonads, liver, and thyroid gland, or act directly on other tissues, such as PRL that stimulates milk production in the breast. To accomplish its key biological function, the pituitary integrates stimulatory and inhibitory signals from the hypothalamus, together with negative feedback cues from the target organs, which together control the pituitary’s pulsatile hormonal output (1, 2).

During organogenesis of the gland, the different endocrine cell types all develop from embryonic stem/progenitor cells (3, 4). After birth, a number of stem cells remain present, located in two separate niches, namely the marginal zone (MZ) that lines the embryonically residual cleft between the IL and AL, and scattered clusters in the AL parenchyma. The pituitary stem cells are marked by expression of the transcription factor SRY-box transcription factor 2 (SOX2) and are mostly quiescent in the homeostatic gland (3–7). However, evidence suggests that they may play a role in pituitary cell remodeling during conditions of altered hormonal needs (such as lactation and stress; reviewed in (8)). Their contribution appears to be situated in amplified differentiation toward the wanted endocrine cells and/or in auto- and paracrine signaling (e.g., via wingless-type MMTV integration site (WNT) ligands) activating proliferation of the stem cells themselves and/or surrounding endocrine (progenitor) cells (8–11).

#### 1.1.2 Pituitary diseases

Because of the pituitary’s central and broad function in the endocrine system and organism, clinical manifestations arising from functional deficiency are serious and wide-ranged. Pituitary pathology may involve hypofunction (hypopituitarism) or hyperfunction (hyperpituitarism) (12).

##### 1.1.2.1 Hypopituitarism

Decline or loss of pituitary function causes deficiency in one or multiple hormones. Hypopituitarism can have a congenital cause due to mutations in genes involved in pituitary development and endocrine cell differentiation, such as *POU class 1 homeobox 1* (*POU1F1*, also known as *PIT1*) and *orthodenticle homeobox 2* (*OTX2*) or can be acquired during life (13–16). Pituitary tumors (see below) are the most prevalent culprit of acquired hypopituitarism, because of their expansive growth compressing the healthy pituitary tissue and impairing local blood flow. Furthermore, their treatment by irradiation or surgical resection also causes damage to the healthy tissue resulting in hypofunction. Additional causes of acquired hypopituitarism include central nervous system infections, radiotherapy of non-pituitary brain tumors and traumatic brain injury. The kind of the hypopituitarism symptoms depends on which hormonal axis is affected. For example, GH deficiency results in muscle weakness and osteoporosis in adults. Hypopituitarism patients are treated with lifelong hormone replacement therapy, meaning supplementation of the declined or missing pituitary and/or target-organ hormones (17–21).

##### 1.1.2.2 Hyperpituitarism

Hyperpituitarism is characterized by excess production of one or more pituitary hormones. The most common cause is a functional pituitary tumor (see below), resulting in clinical symptoms according to which of the hormones is over-produced. For instance, excessive production of GH leads to gigantism in children and acromegaly in adults (12, 22).

##### 1.1.2.3 Pituitary tumors

Although pituitary tumorous lesions are detected in 10-15% of (unselected) people at autopsy, clinically relevant symptomatic cases represent a minority (prevalence of 0.1%) but still account for 10-15% of all intracranial tumors, thereby representing their third most prevalent type (23, 24). Most pituitary tumors arise sporadically, while 5% are familial cases through germline mutations (25–28). Although generally classified as benign (and referred to as adenomas), pituitary endocrine neoplasms exhibit a spectrum of behaviors that are not entirely “benign” but can cause significant morbidity and increased mortality risk, even when they are not metastatic. Therefore, pituitary adenomas were recently renamed to pituitary neuroendocrine tumors (PitNETs) (29). Symptoms result from either the (growing) mass of the tumor itself which can compress healthy pituitary tissue (leading to hypopituitarism) and other neighboring structures (such as the optic chiasm resulting in visual disturbances), or from the hypersecretion of hormone(s) by the tumor (hyperpituitarism) (30). Treatment modalities include surgical removal via the transsphenoidal route, medical treatment (e.g., of PRL-producing prolactinomas with dopamine agonists), and radiation therapy (of recurrent or therapy-resistant tumors) (22, 31).

### 1.2 Pituitary *in vitro* models

Various *in vitro* research models have been applied to unravel the pituitary’s biology. Cell lines such as the mouse corticotrope AtT20 and rat somato-lactotrope GH3 are well-known tools to explore hormone regulation (32). However, cell lines are non-physiological since tumor-derived or immortalized and cultured in two-dimensional (2D) format, and mostly represent only one pituitary cell type (8). Primary 3D culture systems, such as pituitary explants, re-aggregated pituitary cells, and stem cell-derived pituispheres overcome some of these shortcomings (8). However, pituitary explants are encumbered by quickly reached expiry which restricts their experimental possibilities, and their functional/cellular complexity (i.e., containing all pituitary cell types, extracellular matrix (ECM), blood vessels, etc.) makes drawing conclusions for specific cell types challenging (8, 33–36). Aggregates, formed by dissociated AL cells that re-cluster by either gyratory movement (37–39) or gravity in hanging-drop culture (40, 41), reproduce cell-type specificities but have not been highly instrumental to study the pituitary stem cell compartment (8). Pituispheres, that represent clonal “balls” of cells that self-develop from pituitary stem cells and can differentiate into the various pituitary endocrine lineages, only show limited expandability (5–7, 42). In recent years, the state-of-the-art technology of 3D organoids has come to the forefront to overcome several of the above-mentioned shortcomings.

### 1.3 Organoid models

Organoids are 3D cell constructs that self-develop from stem cells, and mimic key biological properties of the respective organ. The originating stem cells can be pluripotent stem cells (PSCs), including embryonic stem cells (ESCs) and induced PSCs (iPSCs), or tissue-specific (epithelial) stem cells (TSCs) (43, 44).

TSC-derived organoids, representing the epithelial compartment of the originating tissue, are established by embedding tissue fragments or dissociated cells in an ECM scaffold (e.g., Matrigel or basement membrane extract (BME)) while providing a stem cell-maintaining culture medium, usually encompassing a WNT pathway-stimulatory factor (such as R-spondin 1 (RSPO1)), a bone morphogenetic protein (BMP)-inhibiting component (typically Noggin), and epidermal growth factor (EGF). Besides these generic factors, each specific tissue requires a somewhat different cocktail of growth factors to achieve optimal organoid development and growth, usually based on knowledge of the specific tissue’s stem cell niche or its embryonic development (43–46).

PSC-derived organoids are established through directed differentiation in which the embryogenic process of the tissue-in-focus is recapitulated. First, the cells are specified toward the tissue’s specific germ layer (endoderm, mesoderm, or ectoderm), followed by induction and maturation toward the wanted tissue type by (sequentially) exposing the cells to specific, embryonically active growth and signaling factors. In this procedure, cells are brought into a 3D configuration through aggregation or embedding in a matrix, in which they self-develop and -organize into a tissue-resembling structure (43–45). PSC-derived organoids are structurally more complex than TSC-resulting organoids because they can contain cell types not only from the epithelial compartment but of all three germ layers, thereby more closely mimicking the whole *in vivo* counterpart, although typically remaining earlier in development than TSC-derived organoids (43–46).

Key advantages of organoid models over classic 2D cell lines include their 3D configuration, as occurring *in vivo*, and their competence to recapitulate key functional and phenotypical properties of the original or targeted tissue (43–45). Moreover, TSC-derived organoids are long-term expandable while showing biological, functional, and genomic stability and can be cryopreserved to generate ‘living’ biobanks (8, 43–46). Besides their major potential in fundamental research, organoids, both from normal and diseased tissue, can be harnessed to powerful tools in clinical research. For example, diseased-tissue organoid biobanks can be employed in drug screening explorations (e.g., to predict patient drug response) while the healthy-tissue organoids can be exploited as resources for regenerative therapy (45–50). When starting from TSCs, a current limitation is that organoids recapitulate the epithelial compartment of the tissue, not the other tissue cell types. Therefore, protocols to set up more complex organoid models (‘assembloids’) are being developed for multiple tissues (e.g., endometrium, cancer), combining stromal/fibroblast and/or endothelial cells with the epithelial organoids (51–54).

## 2 Main: pituitary organoid models

**Figure 1:**
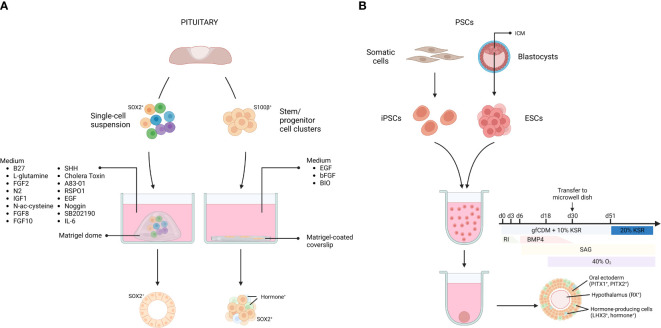
Pituitary organoids derived from primary tissue stem cells or pluripotent stem cells. Organoids from primary pituitary tissue develop from tissue-resident stem cell populations. Single cells or isolated stem cell clusters are embedded in, or overlayed by Matrigel and supplemented with a specific medium **(A)**. Organoids from pluripotent stem cells (PSCs) can be derived from embryonic stem cells (ESCs), established from the blastocyst inner cell mass (ICM), as well as from somatic cell-derived (induced) PSCs (iPSCs). Dissociated PSCs are grown into aggregates and transferred to microwell dishes at day 30. By applying inducing signals in the culture medium, the aggregate differentiates into AL- and hypothalamus-resembling tissue **(B)**. gfCDM, growth factor-free chemically defined medium; KSR, knock-out serum replacement; RI, rock inhibitor; SAG, smoothened agonist. Figure created with BioRender.com. Adapted from (71).

Several organoid systems that have been derived from, or replicate, pituitary tissue, have been established. Below, we give an overview of these 3D organoid models (listed in **Table 1**), either derived from primary pituitary stem cells or from PSCs (**Figure 1**). Subsequently, we describe how these organoid approaches can be used to model and study pituitary diseases.

**Table 1:** Overview of 3D pituitary organoid/spheroid models.

	Tissue/cells of origin	Self-forming?	Expandability?	Hormonal cell types present?	*In vitro* hormone regulation (Treatment: effect on hormone secretion)
Healthy					
(55)	Mouse ESCs	Yes	No	Yes: - ACTH: (secreted) protein, *Pomc* mRNA - GH, PRL, LH, FSH, TSH: protein	Yes: - CRH: ACTH↑ - Glucocorticoid: ACTH↓
(56)	Adult rat S100β^+^ pituitary stem/progenitor cell clusters	Yes	Unknown	Limited: - GH: protein	Unknown
(57)	Human ESCs	Yes	No	Yes: - ACTH, GH: (secreted) protein - PRL, LH, FSH, TSH: protein	Yes: - CRH: ACTH↑ - GHRH + DEXA: GH↑ - GHRH + SS: GH↓
(58)	Adult mouse SOX2^+^ pituitary stem cells	Yes	Limited	Limited: - *In vivo* differentiation: GH, PRL: protein - *In vitro* differentiation: ACTH, αGSU: protein	Unknown
(59)	Human iPSCs	Yes	No	Yes: - ACTH: (secreted) protein, *Pomc* mRNA	Yes: - CRH: ACTH↑ - Low glucose: ACTH↑ - DEXA: ACTH↓
(60)	Adult and aged mouse SOX2^+^ pituitary stem cells	Yes	Extended	No	Unknown
(9)	Neonatal mouse SOX2^+^ pituitary stem cells	Yes	Extended	No	Unknown
Diseased					
Hypopituitarism					
(61)	Patient-derived iPSCs (*OTX2* mutation)	Yes	No	Yes: - ACTH, GH: (secreted) protein	Yes: - CRH: ACTH↑ - GHRH: GH↑
(62)	Patient-derived iPSC (anti-PIT1 hypophysitis)	Yes	No	Yes: - ACTH, GH: protein	Unknown
Tumors					
(63)	Patient-derived corticotropinomas	Yes	Yes	Yes: - ACTH: secreted protein	Unknown
(64)	Patient-derived corticotropinomas	Unknown	Unknown	Yes: - ACTH: secreted protein	- DEXA: ACTH↓
(65)	Patient-derived prolactinoma	Unknown	Unknown	Yes: - PRL: secreted protein	Unknown
(66)	Patient-derived somatotropinomas	Unknown	Unknown	Unknown	Unknown
(67)	Patient-derived pituitary tumors	Yes	No	No	Unknown
	*Drd2* ^-/-^ mouse SOX2^+^ pituitary (tumor) stem cells	Yes	Yes	No	Unknown
(68)	Patient-derived pituitary tumors	Yes	Unknown	Yes: - ACTH: (secreted) protein - GH, PRL, LH, FSH, TSH: protein	Unknown
	Patient-derived iPSCs (*MEN1* mutation; *CDH23* mutation)	Yes	Unknown	Yes: - ACTH: (secreted) protein - GH, PRL, LH, FSH, TSH: protein	Unknown
(69)	Genetically engineered human iPSC (*USP8* mutation, *USP48* mutation)	Yes	Unknown	Yes: - ACTH: (secreted) protein - GH, FSH, LH, PRL: protein	Unknown
(70)	Patient-derived CP tissue	Yes	Unknown	Not applicable	Not applicable

(ESC, embryonic stem cells; CRH, corticotropin-releasing hormone; DEXA, dexamethasone; SS, somatostatin; iPSC, induced pluripotent stem cell; GHRH, growth hormone-releasing hormone; CP, craniopharyngioma).

### 2.1 Pituitary stem cell-derived organoids

In 2016, Yoshida and colleagues reported organoid-like structures forming out of S100β^+^ stem/progenitor cell clusters isolated from rat primary pituitary tissue (56). The dense cell clusters were suspended in medium containing diluted Matrigel and seeded onto Matrigel-coated coverslips. In the presence of fibroblast growth factor 2 (FGF2) and EGF, most clusters (~90%) started to form cavities, resulting in an organoid-like cystic conformation. Upon WNT activation (through the glycogen synthase kinase 3 beta (GSK3B) inhibitor 6-bromoindirubin-3-oxime (BIO)), a mixture of hormone-expressing cells (16% of the total number of cells) was detected in the 3D structures (56) (**Figure 1**). Clonality and self-renewal and -developing capacity of the organoid-like structures was not reported, thereby raising some reservations on their genuine organoid nature according to the current concept (see above (43, 44, 46)) (**Table 1**).

Organoids from pituitary, adhering to the criteria as described above, were reported in 2019 by Cox et al. (58). The adult mouse AL, containing the anterior MZ stem cell niche, was dissociated into single cells which were seeded in a Matrigel dome and cultured in a specified medium, encompassing the typical organoid-culturing components (EGF, Noggin, RSPO1), together with pituitary-specific growth and signaling factors known to be involved in gland development and/or in stem cell regulation in general (e.g., sonic hedgehog (SHH), FGF8, FGF10)) (58, 72) (**Figure 1**). The organoids clonally grow from SOX2^+^ pituitary stem cells and recapitulate the pituitary stem cell phenotype with expression of typical markers (SOX2, E-cadherin (E-CAD) and cytokeratin 8 and 18 (CK8/18)) (58, 72). Intriguingly, differentiation efficiency toward hormone-expressing cells was limited, and found to be most pronounced, although still moderate, following *in vivo* (subrenal) grafting of the organoids in mice (58). These findings suggest that adult pituitary stem cells do not possess high propensity to differentiate. In accordance, lineage tracing has shown that pituitary stem cells generate endocrine progeny especially in the embryonic and neonatal period (3, 4). It is possible that stem cells in the adult pituitary do not (need to) maintain this capacity, given the very low turnover of the adult gland (73). A more pronounced role in auto-/paracrine signaling (e.g., toward hormonal progenitor/precursor or mature cells) than in differentiating to generate new endocrine cells may explain why adult stem cell organoids do not show pronounced differentiation capacity (9–11, 60, 74). However, alternative explanations including the lack of fully optimal conditions for organoid (stem cell) differentiation cannot be dismissed yet. Interestingly, it was discovered that addition of the cytokine interleukin-6 (IL-6) greatly extended the expansion capacity of the organoids, now achievable for over 6 months (**Table 1**) (60, 72).

In addition to recapitulating the pituitary stem cell molecular phenotype, the organoids also replicate functional pituitary stem cell aspects, including their activation status and functionality linked to specific ages (9, 60) and pathological states (58, 60, 67). During the early-postnatal development and maturation phase of the gland, the local stem cells are highly active regarding proliferation, differentiation, and stemness-related pathway expression. In accordance, postnatal contribution to the different (expanding) endocrine cell types is highest in this neonatal period (3, 8, 9, 74, 75). This activation status is reflected in the organoid formation efficiency, which is higher from neonatal than adult AL, the latter containing stem cells that are predominantly quiescent (9). Moreover, the neonatal AL stem cells need much less exogenous growth factors than their adult counterparts to grow into organoids and drive their expansion (e.g., no Noggin, SHH, FGF8 and FGF10 needed), due to higher endogenous expression and activity of several stem cell-related (signaling) pathways in the neonatal pituitary stem cells, as compared to their adult equivalents (9, 58). In contrast, only limited numbers of organoids are established from aging pituitary, thereby reflecting the declined functionality of the stem cells in the aging gland, which is likely due to the process of ‘inflammaging’, a chronic low-grade inflammatory condition that arises in body and organs with advanced age (60, 76, 77). Intriguingly, the old pituitary stem cells regained their functionality in organoid culture (i.e., proper organoid growth and expansion), postulated to be due to their withdrawal from the restrictive inflammatory *in vivo* environment (60). Similar observations have been reported in organoid culturing from other inflammatory diseases (such as intestinal organoids from ulcerative colitis (UC) (78)). Interestingly, restoration of the inflammatory phenotype was achieved by exposing the UC organoids to pro-inflammatory cytokines to reproduce the *in vivo* environment (78).

Certain pituitary perturbations or diseases have been shown to be linked to altered stem cell activity. Local injury in the adult mouse pituitary, as inflicted by endocrine cell-ablation damage, leads to an immediate proliferative reaction of the resident stem cells, resulting in an increased number with higher activation status including stemness gene expression (60, 79–81). This prompt activation was recapitulated by higher organoid formation efficiency when compared to unperturbed (undamaged) gland. Whole-transcriptome sequencing of the organoids uncovered new damage-associated stem cell markers (e.g., *paired related homeobox* (*Prrx) 1*, *Prrx2*) which were also observed *in vivo*, thereby underscoring the translatability of pituitary organoid-based findings (58, 82). Of note, the stem cell reaction to injury is declined in the aging pituitary (likely due to inflammaging, see above), which is again reflected in lower organoid formation efficiency and stemness gene expression from damaged old *versus* young pituitary (60, 81).

Also in tumorigenic pituitary, local stem cells display an activated phenotype (67, 83–86). It has been advanced that the stem cells may contribute to the tumor development and/or growth, be it in a direct or indirect (paracrine) manner (3, 85, 87). Again, enhanced stem cell activity is found to be mirrored in organoid culturing, resulting in higher formation efficiency and expression of markers associated with the disease states (e.g., stemness marker (*Cd44*), chemokines (*C-X-C motif chemokine receptor 6* (*Cxcr6*)), cytokines (*interferon gamma* (*Ifng), Il1b*), and associated Janus kinase (JAK)/Signal transducer and activator of transcription (STAT) and nuclear factor kappa B (NFκB) signaling components (*Stat1/3/4, Jak2, Rel*)) (58, 60, 67).

Taken together, pituitary stem cell-derived organoids provide an exciting and powerful tool, before not available, to grow and expand pituitary stem cells and decipher their phenotype, functionality, and underlying molecular mechanisms in a reliable manner.

### 2.2 PSC-derived pituitary organoids

The pituitary gland is of dual embryonic origin, with the AL derived from the oral ectoderm and the PL from the neural ectoderm. AL development is initiated by thickening of the oral ectoderm (marked by expression of paired-like homeodomain transcription factor (PITX) 1 and PITX2), forming the hypophyseal placode which is strategically located in front of the future ventral diencephalon (VD). The placode invaginates and detaches from the oral ectoderm to form the pituitary primordium, known as Rathke’s Pouch (RP) (71, 88–94). Suga and colleagues applied this knowledge and recapitulated consecutive steps of *in vivo* pituitary development to induce RP development starting from mouse ESCs (55). The cells were first cultured as large floating aggregates (containing 10,000 cells per aggregate) in a defined medium to drive them into oral (*Pitx1^+^, Pitx2^+^)* and neural (hypothalamic, *Rax^+^*) ectoderm. Then, BMP4 was added, since the VD secretes BMP4 during the formation of the hypophyseal placode, indeed favoring a non-neural fate in the ESC aggregates. Simultaneously, a SHH pathway activator (i.e., smoothened agonist (SAG)) was added, since SHH is needed during RP development, being secreted by the oral ectoderm (excluding the RP progenitor region) and the VD. Treatment of the ESC aggregate cultures with SAG increased the expression of the pituitary-specific marker LIM homeobox 3 (LHX3; expression appearing in > 90% of the aggregates), and RP-resembling vesicles started to form. Importantly, in the absence of neural (hypothalamic) ectoderm, vesicle development did not occur, thereby reproducing the knowledge that interplay between the two juxtaposed tissues is critical for RP morphogenesis (55, 88–93). Finally, development of endocrine lineages in the RP-like structures was pursued, again starting from *in vivo* knowledge. Blocking NOTCH signaling in the cultures increased expression of *T-box transcription factor 19* (*Tbx19)* - a transcriptional regulator required for corticotrope differentiation -, and ACTH^+^ cells appeared, all based on the *in vivo* understanding that *Tbx19* expression is inhibited by NOTCH signaling (95, 96). ACTH^+^ cells comprised around 35% of the non-neural cells (which only represent about 11% of total cells) in the aggregates (55). Following exposure to a WNT agonist, expression of *Pit1* was specifically augmented, the transcription factor that is essential in the generation of the lacto-, somato-, and thyrotrope lineages, in line with the knowledge that canonical WNT signaling promotes *Pit1* expression *in vivo* (97). Subsequently, GH^+^ and PRL^+^ cells appeared (around 5% of the non-neural cells) upon glucocorticoid and estradiol exposure, respectively, indeed previously shown to enhance differentiation toward these lineages (98, 99). Very limited (< 3% of non-neural cells) differentiation towards the other endocrine lineages (thyrotropes and gonadotropes) was reported upon treatment of the aggregates with conditioned medium from PA6 stromal cells (55) (**Table 1**; **Figure 1**).

In a follow-up study, the group created similar structures starting from *human* ESCs. Neural hypothalamus and non-neural oral ectoderm fate were induced in the ESC aggregates by exposure to BMP4 and SAG. After 4 weeks, FGF2 treatment led to the formation of LHX3^+^ vesicles (in about 50% of the aggregates), reminiscent of RP structures. While ACTH^+^ corticotropes emerged spontaneously (12% of all PITX1^+^ cells), the PIT1^+^ lineage was induced, although moderately (9% GH^+^, < 2% PRL^+^ and < 1% TSH^+^ of all PITX1^+^ cells), by glucocorticoid treatment, and NOTCH inhibition promoted the gonadotrope fate (efficiency not stated) (57, 100, 101) (**Table 1**), findings that are intriguingly different from the mouse ESC-derived model. Of note, human iPSCs had already before been driven into several pituitary endocrine cell types, although in a 2D monolayer format (102, 103). The efficiency of hormonal differentiation seems higher in this 2D format. Zimmer et al. reported that 80% of the cells express at least one type of hormone transcript after 60 days of differentiation. The most prominent cell type again was the corticotrope cell (55% of all cells express *POMC*), followed by somatotropes (30%). Only few cells expressed *FSHB* and *LHB* (around 3%) (103).

Next, the focus was turned to the hypothalamic compartment of the model, simultaneously aiming at advancing the RP-resembling development since interaction between hypothalamus/VD and RP is important (and essential) in pituitary development (55, 90, 104). Co-induction of hypothalamic tissue and RP in 3D human iPSC aggregates resulted in ‘hybrid’ aggregates. To achieve this, culture conditions were slightly modified (i.e., increasing the knock-out serum replacement (KSR) concentration from 5 to 10% and the BMP4 concentration from 5 to 10 nM, while doubling the number of cells per well). From day 51 onward, the cultures were kept in 35% growth factor-free chemically defined medium (gfCDM), 35% DMEM-based medium with recombinant ciliary neurotrophic factor (CNTF), and 20% KSR. Finally, extending the culture period (from 60 to over 200 days) resulted in hypothalamic neuron-like cells (corticotropin-releasing hormone (CRH)^+^, thyrotropin-releasing hormone (TRH)^+^, antidiuretic hormone (ADH^+^)) emerging in the inner layers of the aggregate, and AL-like cells (LHX3^+^, ACTH^+^) on the outer rim (**Figure 1**) (59, 71) (**Table 1**). The composite organoid model displayed a functional hypothalamic-pituitary axis regarding CRH-ACTH interaction, as illustrated by, among others, decreased ACTH release upon exposure to a CRH receptor inhibitor. Differentiation toward the other pituitary endocrine lineages was not shown (59). Finally, the question was addressed whether the pituitary cell compartment could be enriched from the hybrid PSC-derived organoids (105). Using magnetic-activated cell sorting, epithelial cell adhesion molecule (EPCAM)^+^ cells were isolated that formed aggregates expressing AL markers (PITX1, LHX3, ACTH), present in enriched proportion when compared to unsorted hybrid hypothalamic-pituitary culture (59, 105). EPCAM^-^ cells did not generate AL marker-expressing aggregates (105). It was theorized that sorted cell aggregates could serve as source of stem/progenitor cells and their endocrine descendants. However, the presence of stem cells was not demonstrated (e.g., by immunostaining of SOX2) and so far, only ACTH^+^ cells were shown to develop (105). Taken all these studies together, it appears that differentiation toward corticotropes is the most (and only) efficient path (30% of the obtained organoids contain both LHX3- and ACTH-expressing cells; (71)) presently achieved in these PSC-derived 3D models. Recently, another study attempted to differentiate iPSC-derived pituitary organoids more robustly toward the gonadotrope fate by making some changes to the differentiation protocol (e.g., addition of FGF8, FGF10, and a transforming growth factor-β (TGFβ) inhibitor), but did not succeed (106). Nevertheless, the PSC-derived organoids showed promising *in vivo* production of ACTH. Grafting the organoids in the subrenal capsule of hypophysectomized mice rescued systemic glucocorticoid levels through ACTH secretion in response to CRH loading, as assessed 7-10 days after transplantation (55, 57). Also, subcutaneous grafting of (human ESC-derived) pituitary organoids in hypophysectomized mice resulted in ACTH levels that were consistently higher than in the sham-operated control group for up to 26 weeks post-transplantation (107). The animals showed increased activity in a running wheel test and lost less weight than the control mice which emaciated due to ACTH insufficiency after hypophysectomy. Success after subcutaneous transplantation points to promising translational perspectives.

Taken together, the above-mentioned PSC-derived pituitary organoid models represent interesting tools to explore human pituitary development and interactions with the hypothalamus. However, it should be kept in mind that the set-up of these cultures is labor-intensive and time-consuming, and their expandability disappears once the PSCs have been driven into differentiation. On the other hand, ESCs and iPSCs are readily available, and currently represent the only way to obtain pituitary organoids from human origin.

### 2.3 Organoids to model pituitary diseases

One of the main applications of organoid technology is disease modeling. Here, we give an overview of organoid models of pituitary diseases using the above-mentioned techniques (**Table 1**).

#### 2.3.1 Organoids modeling hypopituitarism

As a model of congenital pituitary hypoplasia (61), patient-derived iPSCs with a mutation in the *OTX2* gene, a transcriptional regulator expressed predominantly in the hypothalamus and important for pituitary development, were subjected to the method of simultaneously inducing hypothalamic and pituitary fate (57, 59, 61) (**Table 1**). The initial tissue specifications (i.e., differentiation toward hypothalamic progenitor cells inside the aggregates and oral ectoderm in the outer layer) were not different between *OTX2*-mutant and control iPSC lines. However, after long-term culture (100 days) endocrine cell types did not appear in the *OTX2*-mutant organoids, whereas GH^+^ and ACTH^+^ cells emerged in the control condition. In accordance, the mutant organoids did not secrete hormones in response to CRH or growth hormone-releasing hormone (GHRH). Absence of LHX3 expression in the oral ectoderm, causing apoptosis in the pituitary progenitor cells, was found to be the root of this impaired AL lineage differentiation potential in *OTX2*-mutant organoids. Correction of the mutation using CRISPR/Cas9 gene editing (i.e., generating an isogenic rescued iPSC line for rescue) reversed the phenotype. In chimeric organoids, created using hypothalamic tissue derived from a control iPSC line and oral ectoderm from the *OTX2*-mutant iPSC line, LHX3 expression in the oral ectoderm remained present, indicating that hypothalamic OTX2 is essential for LHX3 expression in the oral ectoderm compartment (61).

Anti-PIT1 hypophysitis, a syndrome in which PIT1^+^ lineages (GH-, TSH-, and PRL-producing cells) are specifically targeted by PIT1-reactive cytotoxic T lymphocytes resulting in deficiency of these hormones, was also modeled using iPSCs (62). The patient-derived iPSCs were induced into pituitary cells and cultured as described above (57) (**Table 1**). Using the model, authors provided novel insights into the pathogenesis of the disease by showing that the PIT1 protein undergoes antigenic processing and presentation (62), thereby further illustrating the potential of such pituitary disease models.

#### 2.3.2 Organoids modeling pituitary tumors

Over the past decade, many studies have developed organoids from tumors of diverse types of cancer (108–112). The tumor-replicating organoid models are highly instrumental to decipher cancer pathogenesis and can be applied to screen for new (and even personalized) therapies.

To model pituitary tumors in 3D, tumor cell lines have been applied. GH3 cells were subjected to spheroid formation by centrifugation. However, the 3D spheroid conformation was quickly lost, reportedly due to “rapid doubling time and surface interferences” (113, 114). GH3 cells have also been employed in a 3D bioprinting approach (115). Cells were mixed with a gelatin and alginate blend and printed in a grid-like structure arranged in multiple layers, followed by layer crosslinking. Cells were found uniformly distributed in the scaffold and grew into spheroids of various sizes after 6-12 days. The 3D printed model was shown to display several advantages above non-printed 3D and 2D systems, including better growth and survival of the cells with more active cell division and more robust intercellular junctions (115). In another study, culture methods of hanging drop, spheroid suspension, or embedding in (or layering on top of) Matrigel were compared (116). The GH3 and RC-4B/C (rat) pituitary tumor cell lines tested were found to favor the ECM (Matrigel) environment, where cell viability was highest. Although pituitary tumor 3D models using tumor or immortalized cell lines can be interesting to explore basic tumor mechanisms, they do not emulate primary pituitary tumor constellation and (epi-)genetic modifications. Moreover, most available pituitary tumor cell lines are derived from experimental animals, and only few from humans (32, 117).

A first organotypic 3D model of primary human pituitary tumor (prolactinoma) was obtained through aggregation (118). The aggregates could be kept in culture for several (over 3) months and were used to test effects of drugs (such as dopaminergic agonists) on the PRL secretory activity of the prolactinoma cells (118). Another approach cultured primary human pituitary tumor cells in 3D (up to 4 months) in alginate beads (119). Spheroids were derived from prolactinomas (65), GH-overproducing somatotropinomas (66), and ACTH-overproducing corticotropinomas (Cushing’s disease) (64)) (**Table 1**). The enzymatically dissociated tumor cells were embedded in Matrigel and cultured in DMEM with 10% fetal bovine serum (FBS). The following day, drug screening was performed using the formed spheroids, which most likely represent cell aggregates (of which the morphology was not shown in the studies), and effects on PRL, GH, and ACTH secretion were measured (64–66). In all these studies, the obtained pituitary tumor spheroid models do not meet yet the organoid criteria (see above), in particular lacking the demonstration of clonality, expandability, and tumor-phenotype recapitulation through deep characterization (**Table 1**).

Zhang and colleagues strived to develop organoids from corticotrope tumors (63). Cells were embedded in a Matrigel drop on top of a thick Matrigel coating and cultured in an optimized medium containing 10% FBS and typical (pituitary) organoid growth factors, such as EGF, insulin like growth factor-1 (IGF-1), FGF8, triiodothyronine (T3), TRH, and bovine hypothalamus extract. After 5 weeks, the tumor organoids (referred to as tumoroids), shown to contain corticotrope (ACTH^+^), stem/progenitor (SOX2^+^) and intermediate (PITX1^+^, TBX19^+^) cell populations, were moved to spinner flasks, which further increased ACTH production. Comparing primary tumor tissue with matched tumor-derived 2D and 3D cultures documented the importance of 3D organotypic culturing. ACTH secretion was rapidly lost in the 2D culture system, whereas hormone secretion was sustained up to 18 weeks in the 3D tumoroid approach. The 3D cultures were described to be expandable, although methodological details are missing (63) (**Table 1**).

A more recent study succeeded in developing organoids from several different pituitary tumor types (i.e., hormone-producing/functioning as well as non-hormonal/non-functioning tumors) (67) (**Table 1**). Patient samples were dissociated, cells embedded in Matrigel domes and cultured in optimized growth medium (containing, among others, hepatocyte growth factor (HGF) and IL-6). The established organoids recapitulate several of the histological features of the tissue of origin (such as differently sized and shaped nuclei) and show a stem cell phenotype (with expression of stem cell markers SOX2, S100β, SOX9, KRT8/18, TACSTD2, and E-CAD), all as found in the tumor samples. Moreover, the recently reported pituitary tumor-related genes *adhesion molecule with Ig like domain 2* (*AMIGO2), ZFP36 ring finger protein (ZFP36), BTG anti-proliferation factor 1 (BTG1)*, and *discs large MAGUK scaffold protein 5* (*DLG5)* were found not only highly expressed in the tumors, but also in the derived stemness organoids (120). However, expression of the (functioning) tumor’s specific hormone(s) was not observed, indicating that only the stem cell compartment in the tumors grows into the organoids which do not spontaneously form the tumor’s endocrine cell type. As a shortcoming, the tumor-derived organoids could not be expanded, in line with the observation of upregulated expression of pro-apoptotic and hypoxia-induced genes and downregulation of pro-survival genes in the organoids compared to the corresponding primary tissue, as revealed by RNA-sequencing analysis (67). Further optimization of culture conditions is needed to generate robust and tractable pituitary tumor organoid lines. Of note, organoids could also be established from mouse tumorigenic pituitary, i.e., from the *Drd2^-/-^* mouse model (homozygous knockout of the dopamine receptor D2 (DRD2), leading to prolactinoma development in the AL at later age (121)) (67). The organoids recapitulate the activated phenotype of the pituitary stem cells during tumorigenesis (84), regarding formation and growth efficiency and stem cell marker expression (see above). Addition of cytokines that were found to be upregulated in the tumorigenic pituitary stem cells (see above), stimulated organoid growth and expandability even further. Similar to the human pituitary tumor-derived organoids, hormone expression (in this case of PRL) was not observed in the organoid cultures. Thus, the present culture method or organoid medium maintains the stem cells present in the tumor or tumorous pituitary but does not endorse them to differentiate into the tumor’s endocrine cell type (67), and further studies are needed.

Using a slightly adapted protocol (such as use of WNT-conditioned medium and FGF18), organoids were developed from corticotrope tumor samples of Cushing’s disease patients (as well as from other PitNET subtypes: gonadotropinomas, prolactinomas, somatotropinomas, and nonfunctional tumors) (68). Since progressive development neither expandability was shown, it is not clear whether the formed structures developed by expansive cell proliferation or rather by aggregation, and whether they were immediately used for downstream analyses or first expanded for a certain period. In the former case, the 3D structures would still contain primary differentiated cells which, in contrast, are typically lost in expanding organoid cultures. Authors detected ACTH expression in their culture system, and ACTH secretion in the conditioned medium. Flow-cytometric analysis showed the presence of different pituitary lineages (which may originate from the tumor and/or adjacent tissue), as well as enrichment of the TBX19^+^ (corticotrope) lineage and stem cells (marked by SOX2, CXCR4 and CD133), similar to the patient’s tumor (68). In addition, the organoids recapitulated the genetic alterations of the patient’s primary tumor (e.g., single nucleotide variations in *BMP4*, *cadherin related 23 (CDH23)*, *AKT serine/threonine kinase 1* (*AKT1*)), as uncovered by whole-exome sequencing. Finally, the organoids were harnessed into drug screening with small-molecule compounds, showing patient-dependent responses, as also observed in clinical treatment of Cushing’s disease patients. Further deciphering the mode of action of the identified active drugs could reveal new therapeutic targets for Cushing’s disease patients. In a follow-up study, patient-specific responses of these primary tumor- as well as iPSC*^USP8^*/iPSC*^USP48^* (see below) -derived organoids to somatostatin agonists (e.g., pasireotide) and glucocorticoid receptor antagonists (e.g., relacorilant), used in the treatment of Cushing’s disease, were noted (69). The study identified a potential therapeutic use of relacorilant in combination with somatostatin analogs.

Pituitary adenoma-mimicking organoids can also be derived from iPSCs. In this method, iPSCs are first subjected to a directed differentiation schedule in a 2D setting. At day 15 of differentiation, cells are harvested and resuspended in Matrigel and cultured in 3D in the gel domes for an additional 15 days (68, 69). Two approaches to model corticotropinomas from iPSCs have been described. A first method utilized Cushing’s disease patient-derived iPSCs (with familial mutations in *multiple endocrine neoplasia link type 1 (MEN1)* and *CDH23*) (68), while a second approach introduced somatic mutations in genes known to be involved in corticotropinoma tumorigenesis (i.e., *ubiquitin specific peptidase* (*USP) 8* and *USP48* (31, 122)), using CRISPR/Cas9 in iPSCs from healthy donors (69). Both approaches resulted in organoids that showed increased expression (compared to control (CTRL), i.e., non-mutated iPSC*^CTRL^*-derived pituitary organoids) of TBX19 and ACTH, whereas development of other lineages was suppressed (as revealed by decreased expression of PIT1 and hormones such as GH and FSH/LH). Also, increased expression of the PitNET marker synaptophysin (SYPH) was observed as well as an elevated proliferative index (68, 69). Moreover, in the iPSC*^CDH23^*-derived organoids unique proliferative cell populations were revealed using multicolor flow cytometry analysis, including stem/progenitor cell clusters, mesenchymal cells, and endothelial progenitor cells. Authors hypothesized that the presence of these different cell populations might indicate that they support or contribute to the adenoma growth and progression, and that the expanded stem/progenitor cells might be the targets for tumor recurrence (68). The genetically engineered organoids (iPSC*^USP8^*/iPSC*^USP48^*) were used to assess the effects of the glucocorticoid receptor modulators mifepristone and relacorilant. Organoids exhibited divergent responses to these different modulators regarding somatostatin receptor expression, ACTH secretion, and apoptosis/proliferation (69). Hence, such models may have important translational perspectives regarding drug discovery and mode of action identification.

Craniopharyngiomas (CP) represent a subtype of pituitary tumors, most prevalently seen in children, and are believed to originate from embryonic pituitary tissue remnants (123). Recently, an organoid model derived from resected CP tissue was described (70). CP cells were mixed with Matrigel and organoid medium, seeded in ultra-low attachment plates with DMEM/F12 supplemented with B27, EGF, and FGF10, and grown for 30 days. The organoids’ morphology was found similar to that of the primary tumor, and organoids expressed typical CP markers, including cytokeratin 7 (CK7), CD133 and catenin beta 1 (CTNNB1). The organoids were exploited to assess the cell-killing effects of B7-H3-targeted chimeric antigen receptor (CAR) T cells and antibody-DM1 conjugate, revealing that B7-H3 might be a promising target for antibody-drug conjugate therapy against craniopharyngioma (70).

## 3 Conclusion

Over the years, multiple different 3D models of the (anterior) pituitary have been established, both from healthy and diseased conditions. Although often referred to as organoids, the models do not always (strictly) adhere to the ‘organoid’ definition, as described above and, among others, formulated by NCI-NIH as “A tiny, 3D mass of tissue that is made by growing stem cells (cells from which other types of cells develop) in the laboratory. Organoids that are similar to human tissues and organs, or to a specific type of tumor, can be grown” (124).

Organoid technology can also be harnessed into regenerative, cell replacement purposes. Differentiation of pituitary endocrine cell types has been achieved, particularly in the PSC-derived models, although not yet at high efficiency and thus should be further optimized if successful cell replacement is envisioned. Moreover, since organoid culturing from TSCs is in general easier, faster, and less labor-intensive than from PSCs, it would be valuable that efficient differentiation is also achieved in pituitary stem cell-derived organoids. However, it is not clear yet whether the (postnatal) pituitary stem cells have the capacity to efficiently do so or whether they rather play other (auto-/paracrine-regulatory) roles. Another hurdle toward regenerative applications is the current lack of primary pituitary organoids from humans since healthy tissue samples are obviously not easy to come by.

Finally, current organoid-typical models recapitulate the epithelial compartment of the originating tissue. Including other tissue cell types such as endothelial and mesenchymal cells, believed to be an integral part of the pituitary stem cell niche, to establish ‘assembloids’ will further advance the *in vitro* pituitary mimicry. In the end, it would be highly interesting to combine cell/organoid models from different organs of the endocrine axes. Mimicking entire hypothalamus-pituitary-target organ axes would give new insights in how the different entities interact in development and (organismal) functioning in healthy and diseased conditions.

## Author contributions

EL collected all the information and wrote the manuscript. HV co-wrote, critically revised, and finalized the manuscript. Both authors contributed to the article and approved the submitted version.
